# A Call for Action: The Application of the International Health Regulations to the Global Threat of Antimicrobial Resistance

**DOI:** 10.1371/journal.pmed.1001022

**Published:** 2011-04-19

**Authors:** Didier Wernli, Thomas Haustein, John Conly, Yehuda Carmeli, Ilona Kickbusch, Stephan Harbarth

**Affiliations:** 1 Division of International and Humanitarian Medicine, University of Geneva Hospitals and Faculty of Medicine, Geneva, Switzerland; 2 Infection Control Program, University of Geneva Hospitals and Faculty of Medicine, Geneva, Switzerland; 3 Infection Control Unit, Tel Aviv Sourasky Medical Center, Tel-Aviv, Israel; 4 Global Health Programme, Graduate Institute of International and Development Studies, Geneva, Switzerland

## Abstract

Stephen Harbarth and colleagues argue that the International Health Regulations (IHR) should be applied to the global health threat of antimicrobial resistance.

**Summary Points**

- The public health threat of antimicrobial resistance (AMR) is growing and needs to be addressed urgently.
- The International Health Regulations (IHR), a legally binding agreement between 194 States Parties, whose aim is to prevent, protect against, control, and provide a public health response to the international spread of disease, deserve critical examination with regard to their applicability to AMR.
- We argue that the emergence and spread of antimicrobial-resistant bacteria, especially those involving new pan-resistant strains for which there are no suitable treatments, may constitute a public health emergency of international concern (PHEIC) and are notifiable to the World Health Organization under the IHR notification requirement.
- The use of the IHR framework could considerably improve our response to emerging AMR threats like carbapenem-resistant Enterobacteriaceae (CRE).
- As more governments start to take the threat of pan-resistant bacteria seriously, there is a window of opportunity for having a healthy debate about the applicability of the IHR to AMR.

The unrelenting rise of antimicrobial resistance (AMR) constitutes a serious threat to health worldwide. In the last decade, challenging multi-resistant bacteria have expanded while new antimicrobial drug development has lagged [1] with little coordinated containment action at the global level. Of significant concern has been the emergence of vancomycin-resistant *Staphylococcus aureus,* extensively drug-resistant (XDR)-tuberculosis, and carbapenem-resistant Enterobacteriaceae (CRE).

AMR in both humans and animals represents a complex global concern that must be addressed “urgently and aggressively” [2]. The International Health Regulations (IHR), a legally binding agreement between 194 States Parties [3], deserve critical examination with regard to their applicability to AMR. Using the example of CRE as point of departure, we analyze and discuss the potential role of the IHR with respect to AMR.

## The Public Health Risk Posed by CRE

Enterobacteriaceae, a family that includes common pathogens responsible for a large spectrum of disease, have been sensitive to many antibiotics in the past. Since the 1980s, the global spread of extended-spectrum β-lactamase (ESBL)-producing Enterobacteriaceae has limited therapeutic options, but until recently, carbapenems were still a reliable treatment. The recent emergence of CRE, resistant to most classes of antibiotics, has necessitated the use of third-line agents and combination therapy with doubtful therapeutic efficacy and increased toxicity [4].

*Klebsiella pneumoniae* harboring KPC (KPC-Kp) have become endemic in parts of the United States, China, Israel, and Greece [4]. KPC-Kp have been imported from the United States to Israel, and from Israel to Colombia, the United Kingdom, and Greece. International spread of KPC-Kp from Greece has occurred to at least nine European countries since 2007 with further transmission documented in four of them (Table 1 and Figure S1). CRE-producing metallo-β-lactamases of the VIM family have become highly prevalent in Greece since their first detection in 2001 and spread to other countries in Europe and America [5]. NDM-1-producing CRE likely originated in India or Pakistan and have spread to four continents [6],[7].

**Table 1:** Transmission of carbapenem-resistant *Klebsiella pneumoniae* from Greece to other European countries, 2007-2010.

Country	Year	Total Number of Patients	Origin of Patients	Number of Secondary Cases	Probability of the Greek Origin	References	Mechanisms of Resistance
**Belgium**	2009	3	3 patients transferred from Greek hospitals	0	Confirmed	Bogaerts et al. 2010 [19]	*bla*KPC-2
**Denmark**	2009	2	2 patients transferred from Greek hospitals	0	Confirmed	Hammerum et al. 2010 [20]	*bla*KPC-2
**Finland**	2009	1	1 patient transferred from Crete	0	Confirmed	Osterblad et al. 2010 [21]	*bla*KPC-2
**France**	No data	8	1 patient transferred from Crete	7	Confirmed	Naas et al. 2010 [22]	*bla*KPC-2
**France**	2007	1	1 patient transferred from Crete	0	Confirmed	Cuzon et al. 2008 [23]	*bla*KPC-2
**France**	2009	1	1 patient transferred from Greek hospital	0	Confirmed	Barbier et al. 2010 [24]	*bla*KPC-2
**France**	2009	4	1 patient transferred from Greek hospital	3	Confirmed	Kassis-Chikhani et al. 2010 [25]	*bla*KPC-2
**Germany**	2007-2008	9	1 patient treated in Greece	8	Hypothetical	Wendt *et al*. 2010 [26]	*bla*KPC-2
**Hungary**	2008	7	1 patient transferred from Greek hospital	6	Confirmed	Tóth et al. 2010 [27]	*bla*KPC-2
**Norway**	2007	6	4 patients transferred from Greek hospitals	2	Confirmed	Samuelson et al. 2009 [28]	*bla*KPC-2
**Sweden**	No data	1	1 patient transferred from Greek hospital	0	Confirmed	Tegmark Wisell et al. 2007 [29]	*bla*KPC-2
**The Netherlands**	No data	14	African immigrants travelling via Greece	No data	Hypothetical	Meessen et al. 2010 [30]	*bla*KPC-2
**The Netherlands**	No data	1	1 patient transferred from Greek hospital	No data	Confirmed	Cohen Stuart et al. 2010 [31]	*bla*KPC-2

CRE have been associated with increased mortality and morbidity, and higher treatment costs, when compared to infections caused by susceptible strains [8],[9], and have the potential to considerably increase the risk associated with routine medical procedures. Although CRE have emerged in hospitals, they will eventually spread to the community, similar to ESBL-producing Enterobacteriaceae, resulting in untreatable common infections in otherwise healthy individuals. CRE, particularly NDM-1, are already prevalent in the community in India and Pakistan [6].

The alarming spread of CRE is juxtaposed against our failure to develop new effective antimicrobials. The utility of tigecycline is marred by high rates of resistance among CRE [6] and a recent FDA safety warning [10]. The usefulness of colistin, the last drug with reliable in vitro activity, is limited by toxicity, moderate efficacy, and emergence of resistance [6]. Currently, not a single new agent to treat CRE infections is on the horizon. These observations suggest that the international spread of CRE constitutes a “cause for worldwide concern” [11].

## The Shortcomings of Global AMR Surveillance and Control

Surveillance of AMR-pathogens such as CRE is patchy and limited by financial and technical constraints in large parts of the world. In some high-income countries, AMR data are compiled by publicly funded surveillance networks such as EARS-Net, a network of national surveillance systems in Europe, or by pharmaceutical company-sponsored surveys. Informal networks, such as ProMED, also collect information, although selectively and with a considerable time lag. This holds even truer for the scientific literature.

Improving AMR surveillance is one of the key recommendations in a recent report [2]. Without a global early warning system, the spread of AMR often remains unnoticed until a given strain has become endemic. Although data from Israel indicate that the countrywide adoption of enhanced hospital infection control measures was effective in reducing endemic KPC-Kp transmission, early proactive surveillance and containment strategies are more effective and much less costly [12]. In view of the shortcomings of the current patchwork, a coordinated response using a global framework for surveillance and enhanced infection control of CRE and other emerging XDR-pathogens is needed.

## The Potential Role of the IHR

The IHR provide a legal framework for international efforts to contain the risk from public health threats that may spread between countries, including surveillance and global alerts (Articles 5–11), definition of core public health capacities for surveillance and response in all countries (Articles 5, 13), and World Health Organization (WHO) guidance through “standing recommendations” (Articles 16, 53) [3].

**Figure 1:**
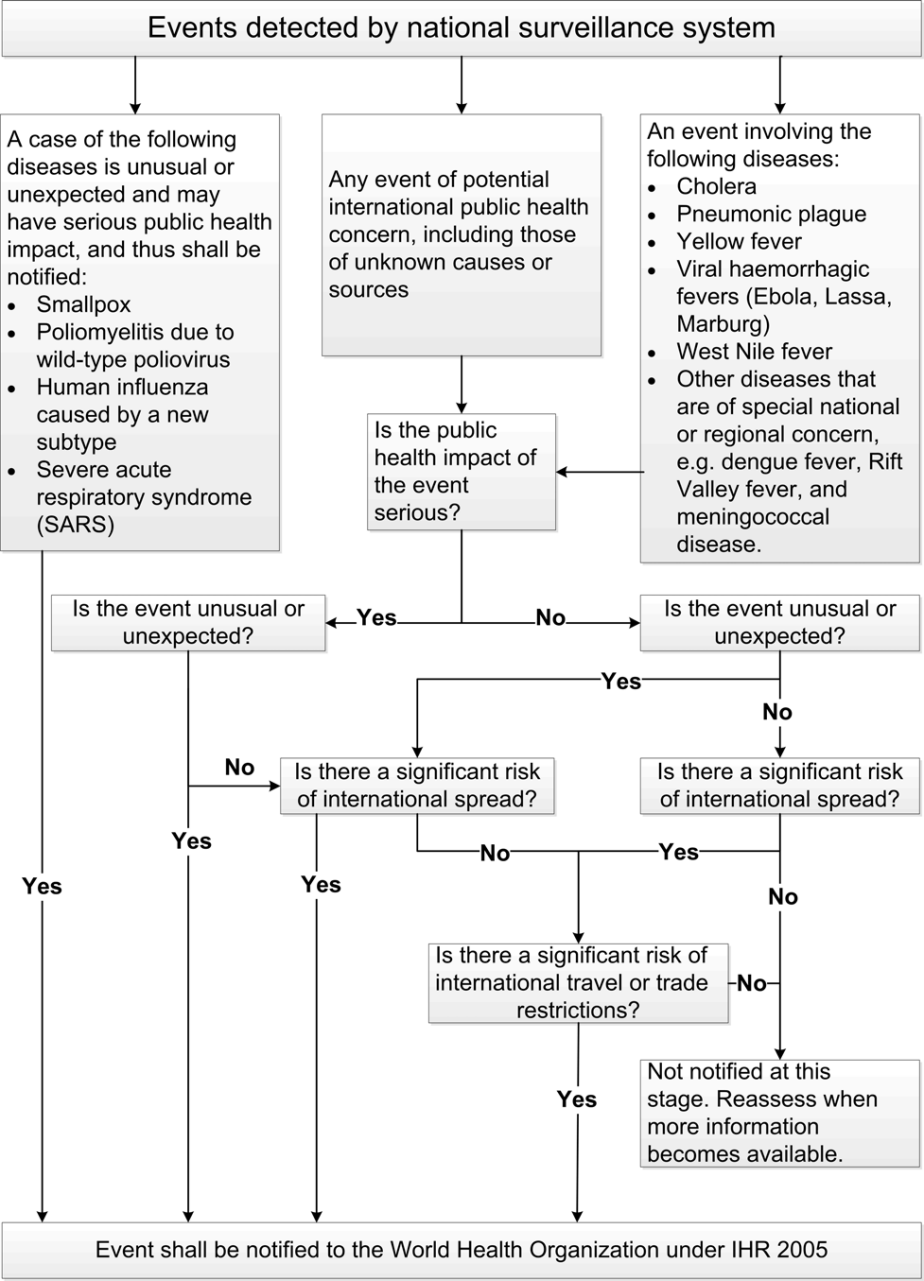
International Health Regulations (IHR) 2005 decision instrument for the assessment and notification of events that may constitute a public health emergency of international concern (simplified from Annex 2 of the IHR).

In order to identify events that have the “potential to cause international disease spread”, WHO is bound to collect epidemiologic information “through its surveillance activities” (Article 5), notifications from affected countries (Article 6), and reports from third parties (Article 9) [3]. A set of criteria defined in Annex 2 of the Regulations (Figure 1) is used to determine whether an event “may constitute a public health emergency of international concern*”* (PHEIC) and “potentially requires a coordinated international response” [3]. The determination of a PHEIC constitutes a second and independent step from the notification process and falls within the purview of the Director-General of WHO.

We argue that certain events marking the emergence and international spread of KPC and NDM-1-producing CRE, especially those involving new pan-resistant strains for which there are no suitable treatments and which are of major public health importance, can be considered to fulfil at least two Annex 2 criteria, in particular “serious public health impact” and “international spread” (Table 2), and should therefore be notified to WHO. This argument has, in fact, been made for XDR-tuberculosis and can be extrapolated to other types of significant new or emerging extensively or pandrug-resistant pathogens such as artemisinin-resistant *Plasmodium falciparum*. “New or emerging antibiotic resistance” is one of the examples listed in Annex 2 for application of the first criterion.

**Table 2:** Arguments in favour of and against the applicability of Annex 2 criteria to new CRE events.

Criterion	Pro	Contra
Is the public health impact of the event serious?	• The spread of CRE has a high potential for future impact on public health. “Public health impact weighs both the immediate and potential future consequences of an event on the health of human populations” [15], although it is not clear whether “future” refers to short-term or long-term consequences.• Treatment failure associated with AMR is one of the “circumstances that contribute to high public health impact” listed in Annex 2 [3].	• Not an immediate threat to public health; short-term impact difficult to quantify. The increased attributable morbidity and mortality is mostly restricted to a minority group, i.e., hospitalized patients. Low potential to cause visible community epidemics compared to infections such as influenza, cholera, or polio.
Is the event unusual or unexpected?	• Novel resistance mechanisms, particularly pan-resistance, are by definition unusual and unexpected.	• Selection of resistant pathogens is an expected consequence of the use of antimicrobials.
Is there any significant risk of international spread?	• Clear epidemiological links and cross-border movement of individuals colonised or infected with CRE [7] (Table 1).	• The international spread of CRE is slow compared to the acute risk to public health caused by respiratory viruses.
Is there any significant risk of international travel or trade restrictions?	• In 2008/2009, Russia refused imports of pork and poultry products based on the presence of antibiotic residues [32]; a similar reaction to the presence of CRE in food items would not seem out of the question in the context of increasing concern about AMR.	• In reality, no case of trade restrictions and no travel restrictions due to CRE so far.

Still, due to the nonspecific nature of Annex 2 and limited WHO guidance, some may counter that CRE (and other AMR) events are irrelevant to the IHR. In a recent survey among National IHR Focal Points, a scenario describing a fatal hospital outbreak caused by pan-resistant *K. pneumoniae* was considered notifiable by just over half of respondents [13]. One of the main arguments against applying the IHR to AMR events is that “the IHR are really intended for outbreaks of acute disease” [14] rather than “acute-on-chronic” events like the relatively slow but relentless spread of AMR. However, we would counter that this reasoning is inconsistent with the explicitly stated purpose of the IHR “to prevent, protect against, control and provide a public health response to the international spread of diseases in ways that are commensurate with and restricted to public health risks, and which avoid unnecessary interference with international traffic and trade”[3].

### Why Should the IHR Be Applied to the Global AMR Threat?

The global threat posed by the spread of AMR cannot be addressed by individual countries alone, but requires a coordinated international response. Recognizing the applicability of the IHR to AMR will serve as a “wake-up call” and strengthen global AMR surveillance and response, which could in turn contribute to containing the spread of AMR. While WHO has initiated several networks and provides guidance for reporting AMR, including WHONET, none function as an early warning system. Although very few AMR events would be determined a PHEIC by the Director-General, notifications of events that fulfil the Annex 2 criteria could serve as alerts and could be an important instrument in the chain of “the global early warning function, the purpose of which is to provide international support to affected countries and information to other countries if needed” [15]. The immediate consequence of notification is to initiate an “exclusive dialogue between the notifying State Party and WHO concerning the event at issue” [15] and to make a joint risk assessment. Once an event has been notified to WHO, and it is not determined to be a PHEIC, WHO can communicate this information to other countries (Article 11). The dissemination of information through the WHO Event Information System (EIS) could expediently increase awareness in multiple countries, allow early implementation of screening measures for persons at risk (e.g., international hospital transfers), and prevent the establishment of new resistant strains in unaffected countries. Based on the experience in Greece and Israel, Carmeli et al. recommend that countries “should be made aware of the problem and should have a preparedness plan ready for implementation at a national level” [16]. By authorizing WHO to make “standing recommendations” (Article 16), the IHR could facilitate the international dissemination of appropriate measures to counter the spread of AMR.

Importantly, the IHR focuses on a societal investment in core surveillance and response capacities at different levels by setting minimum standards. WHO pledges to collaborate with the States Parties concerned “by providing technical guidance and assistance and by assessing the effectiveness of the control measure in place, including the mobilization of international teams of experts for on-site assistance, when necessary”. This is relevant for the spread of AMR given the importance of appropriate infection control measures. While details of these measures need to be more closely defined, it is clear that the application of the IHR framework is invaluable for a coordinated global approach to AMR.

### What Are the Obstacles to Apply the IHR to the Global Spread of AMR?

Even if WHO and a majority of States Parties considered that AMR should be addressed under the IHR, technical, financial, and political obstacles might interfere. Notification of an event to WHO depends on it being detected (requiring a functioning health system and adequate laboratory capacities), and reported to the National IHR Focal Point. There is concern that many States Parties are far from being compliant with the IHR's minimum core capacity requirements for surveillance and response. Even if relevant information filters through to the national level, notification decisions may be under political control. The fierce reaction of the Indian government to claims that NDM-1-producing CRE isolated in the UK originated in India casts doubt on the willingness of governments to report the existence of such events, in particular if economic interests (such as the income from medical tourism) are at stake. These obstacles are not specific to AMR-related events, and cannot serve as an argument against the application of the IHR in this context.

The final obstacles are a lack of expertise and capacities within WHO. Although WHO vertical programs have successfully focused on drug resistance in selected areas, including malaria and tuberculosis, WHO arguably does not have the means to comply with its IHR mandate of offering assistance to States Parties affected by the spread of multi-resistant bacteria. The dearth of leadership in this area was the object of a WHO resolution in 2005, but it has been commented that “very little has taken place to implement the resolution WHA 58.27 since its passage” [17]. During the last World Health Assembly, the Swedish Health Minister commented that “there is an increasing awareness about this major health threat, but far from enough action. The leadership of WHO is urgently needed in this area” [18].

### IHR—A Call for Action

The IHR do not provide a panacea for the problem of AMR. However, this framework provides a global surveillance infrastructure and orchestrates an appropriate public health response. The IHR are ultimately “owned” by the States Parties, some of whom increasingly understand the extent and urgency of the threat posed by AMR. However, it is up to WHO to provide leadership on the role of the IHR in this matter. Further guidance on the application of Annex 2 to this issue is required. With the IHR in place, increasing the capacities of this framework at all levels to address AMR, rather than investing in new vertical programs, seems logical. The revival of the implementation of the WHO 2001 Global Strategy for the containment of AMR with incorporation of the IHR framework into the strategy is required. Although this paradigm shift eventually rests on the World Health Assembly and States Parties' willingness to adopt it, WHO must demonstrate leadership in this regard.

## Conclusion

The international dissemination of AMR, typified by CRE, is a serious threat for global health. Although the spread of AMR is less dramatic than many acute disease outbreaks, it significantly reduces our therapeutic options and adds significantly to the health care burden. A global mechanism incorporating both systematic surveillance and effective public health response is urgently required. We would argue that the IHR provide an appropriate framework to coordinate efforts for controlling the international spread of AMR. Several obstacles need attention before the full potential of the IHR may be realized, but there is a window of opportunity for having a healthy debate about the applicability of the IHR to AMR. While States Parties and WHO share a collective responsibility in the process, WHO must clearly delineate its position with regard to AMR and the intended role of the IHR in this context.

## Supporting Information

Figure S1 Transmission of carbapenem-resistant *Klebsiella pneumoniae* from Greece to other European countries, 2007–2010
(TIF)

## Abbreviations

AMR: antimicrobial resistance
CRE: carbapenem-resistant Enterobacteriaceae
IHR: International Health Regulations
KPC-Kp: *Klebsiella pneumoniae* harboring KPC
PHEIC: public health emergency of international concern
WHO: World Health Organization
XDR: extensively drug-resistant
