# Supplementary figures and images for: A Call for Action: The Application of the International Health Regulations to the Global Threat of Antimicrobial Resistance
Source: PLoS Med. 2011 Apr 19;8(4):e1001022. doi: 10.1371/journal.pmed.1001022 (PMC3079636; doi:10.1371/journal.pmed.1001022)

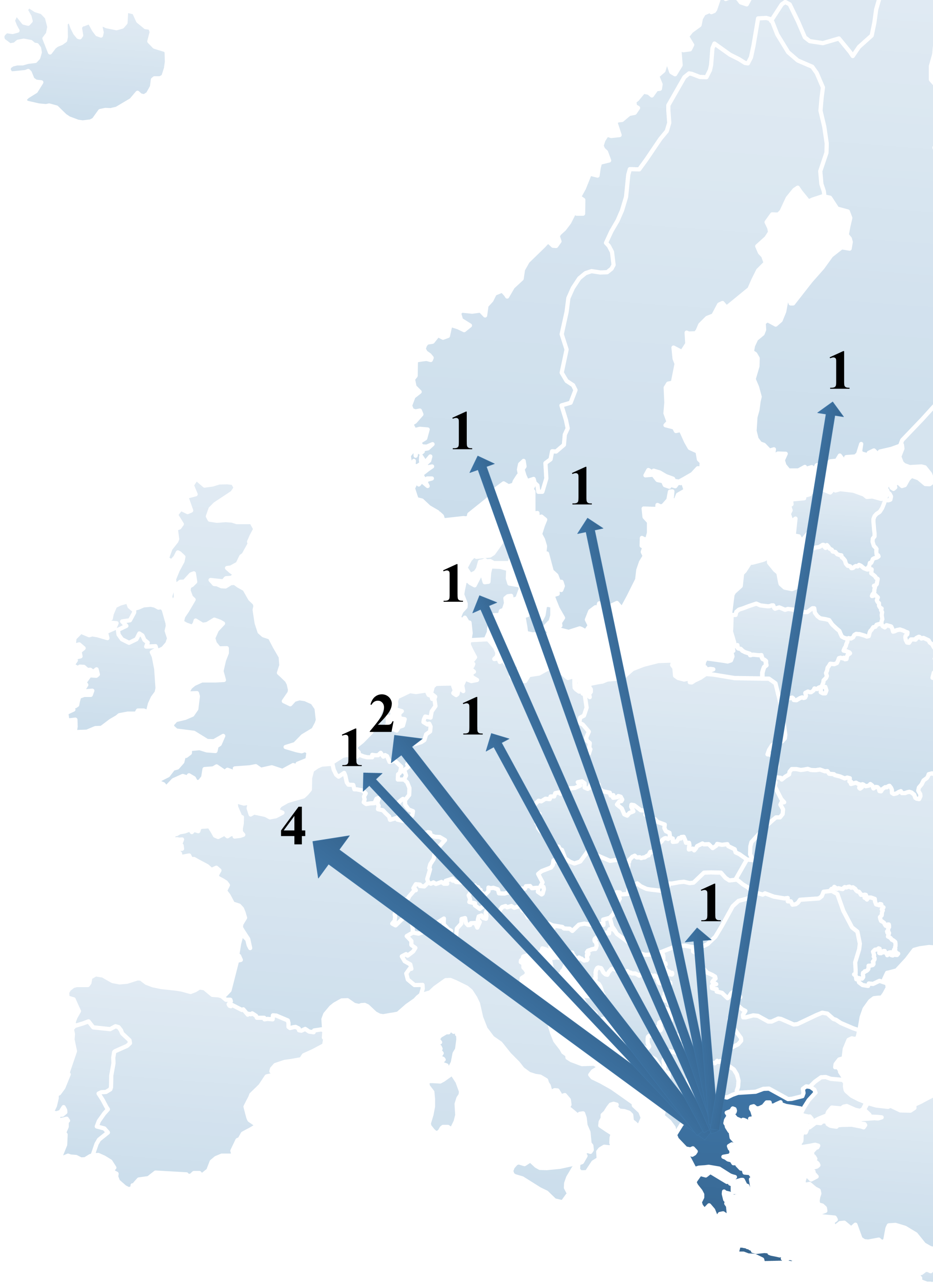

Supplement: Figure S1 — Transmission of carbapenem-resistant Klebsiella pneumoniae from Greece to other European countries, 2007–2010 (TIF) [file pmed.1001022.s001.tif]
